# Health-related quality of life among menopausal women: A cross-sectional study from Pokhara, Nepal

**DOI:** 10.1371/journal.pone.0280632

**Published:** 2023-01-20

**Authors:** Samjhana Baral, Hari Prasad Kaphle

**Affiliations:** School of Health and Allied Sciences, Pokhara University, Pokhara, Gandaki, Nepal; National Academy of Medical Sciences, NEPAL

## Abstract

**Introduction:**

Menopause is the permanent cessation of menstruation. Quality of life is a broad concept affected by an individual’s physical health, psychological state, level of independence, societal relationship, and environmental features. During the menopausal period, women can experience various symptoms affecting their quality of life. This study assesses the factors associated with health-related quality of life among menopausal women.

**Materials and methods:**

A community-based cross-sectional study was carried out among 249 menopausal women to assess their health-related quality of life, associated factors, and self-reported health problems. A pre-tested structured interview schedule was used to conduct face-to-face interviews to obtain the information per the study’s objective. The Menopausal Rating Scale (MRS) was used to assess the health-related quality of life. Data was entered in Epi-data, and analysis was done using the Statistical Package for Social Sciences (SPSS). Univariate, bivariate, and multivariate analyses were carried out to obtain results per our objectives.

**Results:**

The study found that 51.4% of menopausal women had poor quality of life. The mean and standard deviation of the total MRS score was found to be 9.5±5.3. Ultimately, the factors such as educational attainment {Adjusted Odds Ratio (AOR) = 5.779, 95% Confidence Interval (CI): 2.029–16.459}, medication/treatment of the health problems (AOR = 4.828, 95% CI: 1.662–14.023), alcohol intake status (AOR = 8.006, 95% CI: 2.016–31.785) and physical activity (AOR = 5.746, 95% CI: 1.144–28.872) were significant determinants of quality of life among menopausal women.

**Conclusion:**

There is a need to pay proper attention to factors affecting the quality of life to improve the status of menopausal women.

## Introduction

World Health Organization (WHO) defines the Quality of Life (QoL) as “the perceived position of an individual in life in the context of the cultural system and value systems in which they reside and concerning their goals, expectations, standards, and concerns.” Hence, it is a wide-ranging concept affected by an individual’s physical health, state of psychology, independence level, relationships within society, and relationship to salient environmental features which indicates that quality of life is subjective and multi-dimensional [1].

Menopause is defined as the permanent cessation of menstruation due to the loss of ovarian follicular activities and hormone deficiency, causing an adverse effect on the life of some women [2]. Every woman beyond 55/60 experiences a shift from the reproductive to the non-reproductive phase of life; the most apparent feature is the stoppage of menstruation, i.e., Menopause [3]. Globally, the biological age for menopause is 45 to 55 years [4], with an average of 48.7 years in Nepal [5]. During this period, women can experience various symptoms such as hot flashes, sweats at night, sleep and mood disturbance, memory and concentration problems, nervousness and depression, sleeping disorders, bone and joints problems, and reduction of muscle [4], Sexual dysfunction, incontinence, increased fracture, cardiovascular disease, reproductive cancers, osteoporosis, and heart disease were some problems related to Menopause [6, 7].

Menopausal symptoms negatively impact Health-Related Quality of Life (HRQoL) among women [2, 8, 9]. The symptoms connected with poor health-related quality of life could be distressing because they occur when women have essential societal roles, the family, and the workplace [10]. In addition, many women experience symptoms of Menopause, most of which are self-limiting, unpleasant, and sometimes disabling [4]. Hence, the quality of life among menopausal women is essential, and management of menopausal symptoms uplifts the health-related quality of life of menopausal women.

Higher prevalence (40%–60%) of physical, psychological, vasomotor, and sexual disorders among menopausal women and a positive linear relationship between menopausal changes and HRQoL can be found in studies [8, 9]. Among US women, the mean total Menopause Rating Score (MRS) (*n* = 8373) was 11.3±8.5 (median 10); for the somatic subscale, 4.1±3.4; the psychological subscale, 4.6±3.8 and the urogenital subscale, 2.5±2.7. Impaired health-related quality of life (severe total MRS score ≥17) was associated with the use of alternative therapies for menopause, the use of psychiatric drugs, attending a psychiatrist, being postmenopausal, having 49 years or more, and living at a high altitude [11]. Various studies have indicated that Asian women mostly report somatic symptoms than psychological, vasomotor, and sexual symptoms [12]. The somatic, psychological, and urogenital symptoms were higher in rural women than urban women, as revealed by the study from Jammu and Kashmir, India. Urban women lived better health-related quality of life than rural women [13]. Health-related quality of life was rated as worse by the surgical menopause group than by the natural menopause group: the total Menopausal Rating Scale (MRS) scores were much higher for the surgical menopause group (mean = 29.4, SD = 6.7) than for the natural menopause group (mean = 20.7, SD = 6.5) [14].

Age, education, ethnicity [2], longer duration since Menopause, not staying with a partner [15] smoking, diet, exercise, reproductive history belief, and attitude towards menopausal symptoms are factors affecting the health-related quality of life among menopausal women.

This study aims to generate new evidence and assess the health-related quality of life and the factors influencing it among menopausal women in Pokhara Metropolitan.

## Materials and methods

### Study design

The study design was cross-sectional.

### Study method

A quantitative study method was used for this study.

### Study setting

The study was conducted in Pokhara Metropolitan, the capital of Gandaki Province, which lies in the Himalayan region. The quality of life of menopausal women and its associated factors has not been assessed in Pokhara at a community level to date. Pokhara is the country’s largest metropolitan city in terms of area and has the second-largest population. It consists of 33 wards with a total population of 414141, among which 212853 are female, according to the National Population and Housing Census (NPHC) 2011.

### Study population

The study population was women aged 50–59 who have experienced natural Menopause and currently residing in Pokhara Metropolitan of Kaski district.

### Sample size

Based on the previous publication, the prevalence of menopausal symptoms was 87.7% [16].

Using the sampling formula, $n=\frac{z^{2}pq}{d^{2}}$

Here,

n = required sample size

P = 87.7% = 0.877

q = 1-p = 1–0.877 = 0.123

z = z statistical at a 5% level of significance (1.96)

d = allowable error (5%)

Now,

$$n=\frac{1.96^{2}*0.877*0.123}{0.05^{2}}$$

n = 165.76

n ≈ 166

Design Effect = 1.5

Therefore, Total Sample size = 166*1.5 = 249

### Sampling procedure

Multiple methods of sampling were used in the study process. Two wards from Pokhara Metropolitan, i.e., wards 17 and 22, were selected conveniently for this study due to the COVID pandemic as it was impossible to include many wards. However, these two wards consist of diverse populations and represent the Pokhara metropolitan population regarding geography, facilities, and overall living standards. Ward 17 is the biggest ward with the largest population, and ward 22 is the former Village Development Committee. Ward number 17 consists of 26752 population with 13982 females, while ward 22 consists of 7391 population with 4033 females, according to the National Population and Housing Census (NPHC) 2011.

Furthermore, cluster sampling was used for the study. Sampling was done based on the Enumeration Areas (EA) created for the NPHC 2021 conducted by the Central Bureau of Statistics (CBS). Ward number 17 consists of 61 EAs, and ward 22 consists of 7 EAs. Each enumeration area consists of around 200 households on average. Samples were collected based on the proportion of females in these two wards. Accordingly, 193 samples were collected from ward 17, and the remaining 56 were collected from ward 22. Seven enumeration areas were selected from ward 17, and two enumeration areas were selected from ward 22 using a computer-generated random number. In each of the chosen enumeration spots, 30 women aged 50–59 and fulfilling the inclusion criteria were interviewed by house-to-house survey until the sample required for our study was obtained.

### Selection criteria

#### Inclusion criterion

- Women currently living in Pokhara Metropolitan.
- Women aged 50–59 with natural Menopause for at least 12 consecutive months.

#### Exclusion criterion

- Women who were unable and unwilling to answer.

### Ethical considerations

Before conducting the study, ethical approval was obtained from the Institutional Review Committee, Pokhara University. Approval was also taken from the relevant authorities. Both verbal and written informed consent was obtained from participants. The confidentiality and privacy of participants were maintained.

### Research tools and their development

A pre-tested structured interview schedule was used to obtain the information per our objectives. In addition, health-related quality of life was assessed using a standard tool, i.e., the Menopausal Rating Scale (MRS).

The Menopause Rating Scale (MRS) is a health-related quality of life scale developed in Germany (by The Berlin Center for Epidemiology and Health Research) in the early 1990s in response to the lack of standardized scales to measure the severity of menopausal symptoms and their impact on HRQoL. The MRS has been shown to have high reliability, validity, excellent applicability, and sufficiently good repeatability [17].

Health-related quality of life was assessed using the Nepali version of the Menopause Rating Scale (MRS) validated by Gehanath Baral, consisting of 11 items with three dimensions [18].

Dimension 1: Somatic (4 items)

- Hot flashes
- Heart Discomfort
- Sleep problems
- Joint and muscular discomfort

Dimension 2: Psychological (4 items)

- Depressive mood
- Irritability
- Anxiety
- Physical and mental exhaustion

Dimension 3: Urogenital (3 items)

- Sexual problems
- Bladder problems
- Dryness of vagina

Each of the 11 symptoms in MRS contained in the scale can get 0 (no complaints) or up to 4 scoring points (severe symptoms) depending on the severity of the complaints perceived by the women. The somatic domain has a total score ranging from 0 to 16; the urogenital domain has a total score from 0 to 12; the psychological has a total score ranging from 0 to 16. The overall score ranges from 0 to 44. This total score determines the impairment of QoL in the form of no or little (score 0–4), mild (score 5–8), moderate (score 9–16), and severe (score 17–44). Moderate‑to‑severe impairment in QoL was taken as poor QoL for analysis purposes. The scores for each dimension were based on adding the scores of each item of the respective dimensions. The composite score (total score) is the sum of the dimension scores.

The questionnaires on background information and self-reported health problems were developed after an extensive review of related literature and in consultation with the supervisor and research experts.

The tool consisted of five parts as follows:

Part 1: Socio-demographic factors

Part 2: Obstetric and gynecological factors

Part 3: Lifestyle-related factors

Part 4: Health Problems

Part 5: Menopausal Rating Scale (MRS)

### Pretesting, validity, and reliability

Pre-testing was done among similar women in other wards of Pokhara. Health-related quality of life was assessed using a standard tool. The background information was considered using the questions developed by the researcher through an extensive comparative literature review and in consultation with the supervisor and research experts.

### Data collection procedure

A face-to-face interview was carried out to collect the data. A structured interview schedule was developed to obtain the information based on the study’s objective. The eligible women were interviewed after taking both verbal and written consent. The objective and purpose of the research were clearly described before consent. Confidentiality was also maintained. They were assured of voluntary participation. The Nepali language was used to collect the data. The data collection activity was carried out following precautions against COVID-19.

### Data analysis

Participants’ responses were closely examined and recorded in the tool. Data were entered in Epi Data software, and analysis was performed with the help of the Statistical Package for Social Science (SPSS). Univariate, bivariate, and multivariate analyses were done. Frequency distribution and cross-tabulation between dependent and independent variables described and summarized participants’ essential backgrounds and characteristics. Descriptive statistics (i.e., frequency, percentage, mean and standard deviation) were applied to calculate the overall HRQoL. Chi-square and unadjusted odds ratios were identified as a part of the bivariate analysis. Finally, category-wise logistic regression was applied to identify the determinants of quality of life among menopausal women.

## Results

### Univariate analysis

“Table 1” shows the study participants’ socio-demographic, obstetric, and lifestyle-related characteristics, and health problems. Out of 249 participants, more than half (55.0%) were between the age group 55–59, with the mean age and standard deviation of participants as 54.96±2.91. Three-quarters of them (76.3%) were Brahmin/Chhetri, and most (92.4%) followed the Hindu religion. Likewise, more than one-third of the participants (39.8%) were illiterate, followed by non-formal education (21.7%), primary education (18.9%), secondary education (17.7%), and higher education (2.0%) respectively. More than half of them (60.6%) were homemakers, followed by agriculture (18.1%) and business (11.6%). Three-quarters of them (76.7%) were married. Furthermore, most participants (71.9%) were economically dependent, and nearly all (95.6%) were getting family support. One-fourth of them (26.1%) had a monthly family income less than Rs.20000, followed by Rs.20001 to 30000 (23.7%), 40001 to 50000 (21.3%), more than 50000 (18.1%), and 30001 to 40000 (10.8%) respectively. More than half of them (57.4%) experienced menarche at the age of 15 or more. Similarly, most (83.9%) married below the age of 20, and nearly all (98.4%) had been pregnant. Almost half of them (49.4%) had their first pregnancy below the age of 20. Likewise, more than half of them (57.4%) experienced Menopause at the age of 48, and more than one-third (36.9%) faced problems during Menopause. Only 28.9% of them had obstetric and gynecological problems. Similarly, more than half of them (54.2%) were under medication/treatment for the problems. Most participants (78.7%) never smoked, and more than three-quarters of them (88.4%) never drank alcohol. Similarly, most (79.1%) performed household work daily, and very few (8%) did not have physical activity. In addition to that, very few of them (10.8%) practiced yoga and meditation.

**Table 1:** Socio-demographic, obstetric, lifestyle-related characteristics and health problems of the participants (n = 249).

Characteristics	Frequency (n)	Percentage (%)
**Age**		
50–54	112	45.0
55–59	137	55.0
Mean±S.D (54.96±2.91)		
**Ethnicity**		
Brahmin/Chhetri	190	76.3
Janajati	32	12.9
Dalit	26	10.4
Muslim	1	0.4
**Religion**		
Hindu	230	92.4
Buddhist	7	2.8
Christian	11	4.4
Muslim	1	0.4
**Educational attainment**		
Illiterate	99	39.8
Non-formal education	54	21.7
Basic education	47	18.9
Secondary education	44	17.7
Higher education	5	2.0
**Occupation**		
Housewife	151	60.6
Agriculture	45	18.1
Business	29	11.6
Labor and wages	11	4.4
Government job	9	3.6
Private Job	4	1.6
**Marital status**		
Married	191	76.7
Divorced/ Separated & widowed	58	23.3
**Personal economic situation**		
Economic independence	70	28.1
Economic dependence	179	71.9
**Family support**		
Yes	238	95.6
No	11	4.4
**Monthly Family Income (Rs)**		
20001 to 30000	59	23.7
30001 to 40000	27	10.8
40001 to 50000	53	21.3
More than 50000	45	18.1
**Age at menarche**		
<15	106	42.6
≥15	143	57.4
Median = 15		
**Age at marriage**		
<20	209	83.9
≥20	40	16.1
Median = 17		
**Ever been pregnant**		
Yes	245	98.4
No	4	1.6
**Age at first pregnancy (n = 245)**		
>20	121	49.4
≥20	124	50.6
Median = 20)		
**Age at Menopause**		
<48	106	42.6
≥48	143	57.4
Median = 48		
**Problems faced during Menopause**		
Yes	92	36.9
No	157	63.1
**Obstetric and gynecological problems**		
Yes	72	28.9
No	177	71.1
**Medication or treatment of the above problems (n = 72)**		
Yes	39	54.2
No	33	45.8
**Smoking status**		
Never smoked	196	78.7
Current smoker	32	12.9
Past smoker	21	8.4
**Alcohol intake status**		
Never intake	220	88.4
Current intake	21	8.4
Past intake	8	3.2
**Physical activity**		
Exercise >3 times per week	19	7.6
Exercise <3 times per week	13	5.2
Perform household chores daily	197	79.1
No physical activity at all	20	8.0
**Yoga and meditation**		
Yes	27	10.8
No	222	89.2
**Have any health problems at current (**heart diseases, cancer, diabetes, respiratory, musculoskeletal, and gastro-intestinal problems)		
Yes	187	75.1
No	62	24.9

“Table 2” shows the prevalence of menopausal symptoms based on the menopausal Rating Scale (MRS). The majority of them reported joint and muscular discomfort (75.5%) followed by anxiety (67.9%), physical and mental exhaustion (65.0%), hot flashes, sweating (64.3%), sleep problems (60.6%), depressive mood (59.0%), irritability (58.6%), heart discomfort (53.8%), bladder problems (47.0%), dryness of vagina (41.0%) and sexual problems (35.3%) respectively. The symptoms under the somatic subscale were present among 94.8%, with the mean score and standard deviation being 4.3±2.6 out of 16 (Median 4). Similarly, the symptoms under the psychological subscale were present among 93.2% of them, with the mean score and standard deviation being 3.5±2.4 out of 16 (Median 3). Likewise, the symptoms under the urogenital subscale were present among 65.9%, with the mean score and standard deviation being 1.7±1.7 out of 12 (Median 1). The mean and standard deviation of the total MRS score was 9.5±5.3 (Median 9) out of the total score, i.e., 44.

**Table 2:** Information regarding menopausal symptoms among menopausal women using the Menopausal Rating Scale (MRS).

Characteristics	Frequency (n)	Percentage (%)	Mean±S.D
**Somatic subscale**	236	94.8	4.3±2.6 (Median 4)
Hot flashes, sweating	160	64.3	1.0±0.1
Heart discomfort	134	53.8	0.8±0.9
Sleep problems	151	60.6	1.0±1.0
Joint and muscular discomfort	188	75.5	1.6±1.2
**Psychological subscale**	232	93.2	3.5±2.4 (Median 3)
Depressive mood	147	59.0	0.8±0.8
Irritability	146	58.6	0.9±0.10
Anxiety	169	67.9	0.9±0.8
Physical and mental exhaustion	162	65.0	0.8±0.7
**Urogenital subscale**	164	65.9	1.7±1.7 (Median 1)
Sexual problems	88	35.3	0.4±0.6
Bladder problems	117	47.0	0.8±1.0
Dryness of vagina	102	41.0	0.6±0.8
Total Score			9.5±5.3 (Median 9)

“Table 3” shows the prevalence of poor and good quality of life among the participants. Around half of them (51.4%) had poor quality of life with a total MRS score greater or equal to 9, whereas the remaining 48.6% had a good quality of life with a total MRS score less than 9.

**Table 3:** Quality of life among menopausal women using the Menopausal Rating Scale (MRS).

Characteristics	Frequency (n)	Percentage (%)
Quality of Life		
Poor (Total MRS score≥9)	128	51.4
Good (Total MRS score<9)	121	48.6

### Bivariate analysis

“Table 4” shows the association of socio-demographic, obstetric, lifestyle-related variables and health problems of the participants with quality of life. The result depicts that ethnicity (χ^2^ = 8.480, p-value = 0.014), educational attainment (χ^2^ = 29.703, p-value = <0.001), occupation (χ^2^ = 16.370, p-value = 0.003), marital status (χ^2^ = 4.645, p-value = 0.031), personal economic situation (χ^2^ = 9.598, p-value = 0.002), family support (χ^2^ = 4.261, p-value = 0.039) and monthly family income (χ^2^ = 13.515, p-value = 0.009) were the socio-demographic variables significantly associated with quality of life. Obstetric variables such as age at marriage (χ^2^ = 6.819, p-value = 0.009), age at first pregnancy (χ^2^ = 5.029, p-value = 0.025) and medication/treatment of the problems (χ^2^ = 8.795, p-value = 0.003) were found to be significantly associated with quality of life. Lifestyle-related variables such as smoking status (χ^2^ = 14.015, p-value = 0.001), alcohol intake status (χ^2^ = 19.223, p-value = <0.001), physical activity (χ^2^ = 10.071, p-value = 0.018), yoga and meditation (χ^2^ = 5.749, p-value = 0.017) were found to be significantly associated with quality of life. Health problems at the time of the study (χ^2^ = 5.327, p-value = 0.021) were found to be significantly associated with quality of life among menopausal women.

**Table 4:** Association of socio-demographic, obstetric, lifestyle-related variables and health problems of the participants with quality-of-life categories.

Variables	Health-related Quality of Life		Chi-square value	df	P-value
	Good	Poor			
	n (%) 121 (48.6)	n (%) 128 (51.4)			
**Age**					
50–54	58(51.8)	54(48.2)	0.830	1	0.362
55–59	63(46.0)	74(54.0)			
**Ethnicity**					
Brahmin/Chhetri	99(52.1)	91(47.9)			
Janajati	16(50.0)	16(50.0)	8.480	2	0.014*
Dalit and others	6 (22.2)	21(77.8)			
**Religion**					
Hindu	112(48.7)	118(51.3)	0.012	1	0.911
Others	9(47.4)	10(52.6)			
**Educational attainment**					
Illiterate	33(33.3)	66(66.7)			
Non-formal education	24(44.4)	30(55.6)	29.703	4	<0.001*
Basic education	25(53.2)	22(46.8)			
Secondary education	36(81.8)	8(18.2)			
Higher education	3(60.0)	2(40.0)			
**Occupation**					
Service	11(84.6)	2(15.4)			
Business	17(58.6)	12(41.4)			
Housewife	76(50.3)	75(49.7)	16.370	4	0.003*
Agriculture	15(33.3)	30(66.7)			
Labor and wages	2(18.2)	9(81.8)			
**Marital status**					
Married	100(52.4)	91(47.6)	4.645	1	0.031*
Divorced/ Separated & widowed	21(36.2)	37(63.8)			
**Personal economic situation**					
Economic independence	45(64.3)	25(35.7)			
Economic dependence	76(42.5)	103(57.5)	9.598	1	0.002*
**Family support**					
Yes	119(50.0)	119(50.0)	4.261	1	0.039*
No	2(18.2)	9(81.8)			
**Monthly family income (Rs)**					
Less than 20000	23(35.4)	42(64.6)			
20001 to 30000	25(42.4)	34(57.6)			
30001 to 40000	15(55.6)	12(44.4)	13.515	4	0.009*
40001 to 50000	27(50.9)	26(49.1)			
More than 50000	31(68.9)	14(31.1)			
**Age at menarche**					
<15	55(51.9)	51(48.1)	0.801	1	0.371
≥15	66(46.2)	77(53.8)			
**Age at marriage**					
<20	94(45.0)	115(55.0)	6.819	1	0.009*
≥20	27(67.5)	13(32.5)			
**Ever been pregnant**					
Yes	119(48.6)	126(51.4)	-	1	1.00 (fisher)
No	2(50.0)	2(50.0)			
**Age at first pregnancy(n = 245)**					
<20	50(41.3)	71(58.7)	5.029	1	0.025*
≥20	69(55.6)	55(44.4)			
**Age at Menopause**					
<48	52(49.1)	54(50.9)	0.016	1	0.900
≥48	69(48.3)	74(51.7)			
**Problems faced during menopause**					
Yes	40(43.5)	52(56.5)	1.529	1	0.216
No	81(51.6)	76(48.4)			
**Obstetric & gynecological problems**					
Yes	31(43.1)	41(56.9)	1.244	1	0.265
No	90(50.8)	87(49.2)			
**Medication or treatment of the above problems (n = 72)**					
**Yes**	23(59.0)	16(41.0)	8.795	1	0.003*
**No**	8(24.2)	25(75.8)			
**Smoking status**					
Never smoked	107(54.6)	89(45.4)			
Current smoker	10(31.2)	22(68.8)	14.015	2	0.001*
Past smoker	4(19.0)	17(81.0)			
**Alcohol intake status**					
Never intake	118(53.6)	102(46.4)	19.223	1	<0.001*
Past/current intake	3(10.3)	26(89.7)			
**Physical activity**					
Exercise >3 times per week	15(78.9)	4(21.1)			
Exercise <3 times per week	7(53.8)	6(46.2)	10.071	3	0.018*
Perform household chores daily	93(47.2)	104(52.8)			
No physical activity at all	6(30.0)	14(70.0)			
**Yoga and meditation**					
Yes	19(70.4)	8(29.6)	5.749	1	0.017*
No	102(45.9)	120(54.1)			
**Health problems at current**					
Yes	83(44.4)	104(55.6)	5.327	1	0.021*
No	38(61.3)	24(38.7)			

*Statistically significant at p<0.05

“Table 5” shows the participants’ socio-demographic, obstetric, lifestyle-related factors, and health problems associated with quality-of-life categories. For example, Brahmin/Chhetri women were nearly four times (OR = 3.808, 95% CI: 1.471–9.854) more likely to have a good quality of life than women from Dalit and other ethnic groups. Similarly, Janajati women were three and half times (OR = 3.500, 95% CI: 1.118–10.962) more likely to have a good quality of life than women belonging to Dalit and other ethnic groups.

**Table 5:** Socio-demographic, obstetric, lifestyle-related factors, and health problems associated with quality-of-life.

Variable	UOR	95% CI	P-value
**Ethnicity**			
Brahmin/Chhetri	3.808	1.471–9.854	0.006*
Janajati	3.500	1.118–10.962	0.031*
Dalit and others	1		
**Educational attainment**			
Illiterate	1		
Non-formal education	1.600	0.810–3.159	0.176
Basic education	2.273	1.118–4.619	0.023*
Secondary education	9.000	3.761–21.539	<0.001*
Higher education	3.000	0.478–18.839	0.241
**Occupation**			
Service	24.750	2.886–212.229	0.003*
Business	6.375	1.163–34.934	0.033*
Housewife	4.560	0.953–21.810	0.057
Agriculture	2.250	0.431–11.748	0.336
Labor and wages	1		
**Marital status**			
Married	1.936	1.056–3.550	0.033*
Divorced/ Separated & widowed	1		
**Personal economic situation**			
Economic independence	2.439	1.377–4.321	0.002*
Economic dependence	1		
**Family support**			
Yes	4.500	0.952–21.267	0.058
No	1		
**Monthly family income**			
Less than 20000	1		
20001 to 30000	1.343	0.651–2.771	0.425
30001 to 40000	2.283	0.915–5.691	0.077
40001 to 50000	1.896	0.904–3.977	0.090
More than 50000	4.043	1.798–9.093	0.001*
**Age at marriage**			
<20	1		
≥20	2.541	1.242–5.197	0.011*
**Age at first pregnancy (n = 245)**			
<20	1		
≥20	1.781	1.074–2.956	0.025*
**Medication or treatment of the problems (n = 72)**			
Yes	4.492	1.619–12.461	0.004*
No	1		
**Smoking status**			
Never smoked	5.110	1.659–15.737	0.004*
Current smoker	1.932	0.516–7.239	0.329
Past smoker	1		
**Alcohol intake status**			
Never intake	10.026	2.948–34.100	<0.001*
Past/current intake	1		
**Physical activity**			
Exercise >3 times per week	8.750	2.032–37.671	0.004*
Exercise <3 times per week	2.722	0.638–11.610	0.176
Perform household chores daily	2.087	0.770–5.652	0.148
No physical activity at all	1		
**Yoga and meditation**			
Yes	2.794	1.174–6.651	0.020*
No	1		
**Health problems in current**			
Yes	1		
No	1.984	1.103–3.568	0.022*

*Statistically significant at p<0.05

Similarly, women who received primary education were twice (OR = 2.273, 95% CI: 1.118–4.619) more likely to have a good quality of life than illiterate women. In addition, women who received secondary education were nine times (OR = 9.000, 95% CI: 3.761–21.539) more likely to have a good quality of life than illiterate women.

Moreover, women involved in government or private service were nearly twenty-five times (OR = 24.750, 95% CI: 2.886–212.229) more likely to have a good quality of life than women working for labor and wages. Likewise, women involved in business were almost six and half times (OR = 6.375, 95% CI: 1.163–34.934) more likely to have a good quality of life than women working for labor and wages.

Also, married women were almost two times (OR = 1.936, 95% CI: 1.056–3.550) more likely to have a good quality of life than divorced/separated and widowed women.

Economically independent women were about two and half times (OR = 2.439, 95% CI: 1.377–4.321) more likely to have a good quality of life than economically dependent women.

Furthermore, women with a monthly family income of more than 50000 were four times (OR = 4.043, 95% CI: 1.798–9.093) more likely to have a quality of life than women having a monthly family income of less than 20000.

Women who married at the age of 20 or older were twice (OR = 2.541, 95% CI: 1.242–5.197) more likely to have a good quality of life compared to women who married below the age of 20.

Similarly, women who had their first pregnancy at the age of 20 or greater were nearly two times (OR = 1.781, 95% CI: 1.074–2.956) more likely to have a good quality of life than women who had their first pregnancy below the age of 20.

Likewise, women with medication or treatment of the problems were almost four and half times (OR = 4.492, 95% CI: 1.619–12.461) more likely to have a good quality of life than women without medication or treatment of the problems.

Women who never smoked were five times (OR = 5.110, 95% CI: 1.659–15.737) more likely to have a good quality of life compared to past smokers women.

Similarly, women who never intake alcohol were ten times (OR = 10.026, 95% CI: 2.948–34.100) more likely to have a good quality of life than past/current alcohol intake.

Likewise, women who practiced exercise more than three times a week were nearly nine times (OR = 8.750, 95% CI: 2.032–37.671) more likely to have a good quality of life than women without any physical activity at all.

In addition, women who practiced yoga and meditation were nearly three times (OR = 2.794, 95% CI: 1.174–6.651) more likely to have a good quality of life compared to women who did not practice yoga and meditation. Women who did not have health problems at the time of the study were almost two times (OR = 1.984, 95% CI: 1.103–3.568) more likely to have a good quality of life than women who had health problems at the time of the study.

### Multivariate analysis

“Table 6” shows the predictors of quality of life among menopausal women by multivariate analysis. An adjusted odds ratio was obtained by entering all the independent variables under different categories significantly associated with the chi-square test using the enter method in binary logistic regression analysis. For example, on multivariate analysis, women with secondary education were more likely (AOR = 5.779, 95% CI: 2.029–16.459) to have a good quality of life compared to illiterate women. Similarly, women who had taken medication or treatment for the problems were more likely (AOR = 4.828, 95% CI: 1.662–14.023) to have a good quality of life compared to women who hadn’t taken medication or treatment. Furthermore, women who never intake alcohol were likelier (AOR = 8.006, 95% CI: 2.016–31.785) to have a good quality of life than women who had past/current alcohol intake practice. In addition, women who performed the exercise more than three times a week were more likely (AOR = 5.746, 95% CI: 1.144–28.872) to have a good quality of life compared to women who had no physical activity at all.

**Table 6:** Multivariate analysis of predictors of quality of life among menopausal women.

Variable	AOR	95% CI	P-value
**Socio-demographic factors**			
**Ethnicity**			
Brahmin/Chhetri	2.123	0.702–6.420	0.183
Janajati	2.777	0.765–10.088	0.121
Dalit and others	1		
**Educational attainment**			
Illiterate	1		
Non-formal education	1.255	0.589–2.671	0.556
Basic education	1.996	0.888–4.486	0.094
Secondary education	5.779	2.029–16.459	0.001*
Higher education	0.331	0.020–5.568	0.443
**Occupation**			
Service	9.933	0.462–213.556	0.142
Business	1.277	0.184–8.859	0.805
Housewife	2.679	0.461–15.520	0.273
Agriculture	0.915	0.143–5.866	0.926
Labor and wages	1		
**Marital status**			
Married	1.460	0.726–2.936	0.289
Divorced/ Separated & widowed	1		
**Personal economic situation**			
Economic independence	1.668	0.711–3.912	0.240
Economic dependence	1		
**Family support**			
Yes	3.816	0.639–22.790	0.142
No	1		
**Monthly family income**			
Less than 20000	1		
20001 to 30000	0.937	0.414–2.119	0.875
30001 to 40000	0.970	0.341–2.762	0.954
40001 to 50000	0.726	0.300–1.755	0.477
More than 50000	1.537	0.576–4.105	0.391
**Obstetric, gynecological, and health problems-related factors**			
**Age at marriage**			
<20	1		
≥20	1.595	0.274–9.288	0.603
**Age at first pregnancy (n = 245)**			
<20	1		
≥20	1.146	0.381–3.454	0.808
**Medication or treatment of the above problems (n = 72)**			
Yes	4.828	1.662–14.023	0.004*
No	1		
**Health problems in current**			
Yes	1		
No	2.922	0.733–11.644	0.129
**Lifestyle-related factors**			
**Smoking status**			
Never smoked	3.263	0.980–10.868	0.054
Current smoker	3.433	0.794–14.841	0.099
Past smoker	1		
**Alcohol intake status**			
Never intake	8.006	2.016–31.785	0.003*
Past/current intake	1		
**Physical activity**			
Exercise >3 times per week	5.746	1.144–28.872	0.034*
Exercise <3 times per week	1.935	0.374–10.012	0.431
Perform household chores daily	1.708	0.588–4.965	0.326
No physical activity at all	1		
**Yoga and meditation**			
Yes	1.641	0.597–4.508	0.337
No	1		

*Statistically significant at p<0.05

## Discussion

This study was conducted to assess the quality of life and its associated factors among the menopausal women in Pokhara using the Menopausal Rating Scale (MRS). In the present study, the mean age at Menopause was 47.88±3.2, which is in line with the findings of earlier studies from different parts of Nepal like Kapilvastu (46.3) [19], Kathmandu, Bhaktapur, Lalitpur (48.7) [5], Rupandehi (46.81) [20], Dharan (47.14±4.38) [15], Kathmandu (49.9) [21] and Kaski (47.12±4.34) [22].

The current study reported that around half (51.4%) of menopausal women have a poor quality of life. A similar finding was found in another study where 50.6% had impaired quality of life [23]. Similarly, another study in Ecuador revealed that 53% had a severe total MRS score greater than or equal to 17, presenting an impaired quality of life [24]. However, contrasting findings were obtained in studies from India, where more than 70% of menopausal women had poor quality of life [16]. Similarly, the poor quality of life among menopausal women varied in different studies, with more than 37% of them having poor quality of life in Urban Puducherry, India [25], and more than 65% of them having an impaired quality of life in Bhubaneswar, India [26]. This variation in results can be attributed to the cultural, socio-economic, geographical, and methodological differences between the study settings.

The mean total MRS score of this study was found to be 9.5±5.3, which is lower than the mean score obtained from Ecuador (18±10.6) [24], Haryana, India (12.07±6.2) [16] Bhubaneswar, India (20.42±7.56) [26] and Iran (12.45±7.20) [27] and slightly higher than the study conducted on Egypt (9.11±5.76) [28] and Ecuador (9.1±6.4) [23]. Similarly, a study from Srilanka (10.98±6.90) [29] and America (11.3±8.5) [11] showed a mean total MRS score a bit higher than the current study. This study presented the mean scores of somatic, psychological, and urogenital subscales to be 4.3±2.6, 3.5±2.4, and 1.7±1.7, respectively. However, variation was found regarding the subscale score in many studies from Ecuador (S-score: 7.2±4.5, P-score: 6.9±4.8, U-score: 3.9±3.4) [24], Egypt (S-score: 4.12±2.22, P-score: 2.86±2.50, U-score: 2.13±1.04) [28], India (S-score: 8.24±3.13, P-score: 7.2±3.08, U-score: 4.98±2.21) [26], Iran (P-score: 4.90±3.45, U-score: 3.10±2.46) [27] Srilanka (S-score: 5.16±3.01, P-score: 4.03±3.22, U-score: 1.77±2.21) [29], Ecuador (S-score: 4.0±2.7, P-score: 3.0±2.8, U-score: 2.1±2.5) [23], and America (S-score: 4.1±3.4, P-score: 4.6±3.8, U-score: 2.5±2.7) [11]. The variation might be due to socio-economic status, study setting, and methodological differences.

The frequently experienced symptoms from the MRS were joint and muscular discomfort (75.5%) which is supported by many previous studies [24, 28, 30] followed by anxiety, and physical and mental exhaustion, which is also in agreement with the previous study [26].

Findings from bivariate analysis highlighted that the factors affecting the quality of life showed statistical significance with ethnicity, educational attainment, occupation, marital status, personal economic situation, family support, monthly family income, age at marriage, age at first pregnancy, medication or treatment of the health problems, smoking status, alcohol intake status, physical activity, yoga, and meditation and lastly health problems at the time of the study.

Our study showed no statistical association between age and quality of life, in contrast with the study conducted in India that showed that impaired quality of life was associated with younger age [26]. The variation might be due to the difference in the age range of the included participants.

The present study showed a significant association between ethnicity and quality of life among menopausal women, which is supported by the existing studies from Nepal [15, 31].

The findings revealed that those women who had formal education were more likely to have a good quality of life, which is in line with the study from Iran [32] America [33] and Finland [34] that showed the quality of life of the most highly educated women was more likely to improve than among the less educated ones.

Moreover, the current study also reported that employed women, either government or private, and independent women with self-income were more likely to have a good quality of life, which is supported by the earlier studies from North India [35], America [33] and Iran [32].

Also, married women were more likely to have a good quality of life, similar to studies that showed marital status to be significantly associated with quality of life [28, 36–39]. However, a study showed that marital status did not affect QOL [32]. The contradictory result might be due to the educational level, job opportunities, economic independencies, and higher income level.

Furthermore, the current study revealed that the women with higher monthly family income were more likely to have a good quality of life, which is in line with previous studies that showed women from higher income levels reported better overall health [33, 40]. A previous study showed socio-economic status to be significant in quality of life [41].

In the present study, smoking was significantly associated with the quality of life among menopausal women. Previous studies support that smoking affects the quality of life [42] and is a risk factor [43]. Furthermore, never smoked women had significantly lower scores indicating better quality of life [2].

Similarly, women who never intake alcohol were more likely to have a good quality of life which is supported by the study that revealed alcohol user women had a higher risk of impaired quality of life [25].

Furthermore, women who practiced exercise were more likely to have a good quality of life, which is supported by the findings in England [44] that regularly active women reported better health-related quality of life scores than those who were not regularly active. The more daily time allocated for physical activity, the less the severity of menopausal symptoms and, ultimately, improved quality of life [45].

The current study revealed that the presence of health problems was associated with quality of life among menopausal women, which agrees with existing findings that showed menopausal women with health problems, particularly chronic health problems, negatively affect the quality of life [25, 43, 46, 47].

In multiple logistic regression analyses, factors such as educational attainment, medication/treatment of the health problems, alcohol intake status, and physical activity were found to be significant in quality of life.

The current study’s finding on alcohol intake as a significant predictor of quality of life among menopausal women is supported by the previous research that showed current alcohol/tobacco users as a major determinant of poor quality of life [25].

The current study revealed physical activity as one of the important predictors of quality of life among menopausal women, which is supported by the study from Brazil, which indicated that the women who maintained their total habitual physical activity to more than 60 minutes per day had reduced menopausal symptoms and improved quality of life [45].

Similarly, educational attainment was also found to be a significant factor in the quality of life among menopausal women, which agrees with the study from Egypt that showed academic level to be one of the most significant predictors of menopausal quality of life [28].

## Conclusions

Quality of life was poor, with a cut-off score of 9 or more on about half of the menopausal women of Pokhara.

Factors such as ethnicity, educational attainment, occupation, marital status, personal economic situation, family support, monthly family income, age at marriage, age at first pregnancy, medication or treatment of the health problems, smoking status, alcohol intake status, physical activity, yoga and meditation and lastly health problems at the time of the study were significantly associated with quality of life in bivariate analysis.

Multivariate analysis showed that educational attainment, medication or treatment of the health problems, alcohol intake status, and physical activity were the major factors for quality of life among menopausal women while applying multiple logistic regression.

To conclude, the result supports that menopause causes somatic, psychological, and urogenital problems, and it is associated with educational attainment, medication for health problems, alcohol intake status, and physical activities; however, awareness and intervention are essential to improve health-related quality of life among menopausal women.

### Limitations

The study might lack generalizability. In addition, recall bias is probable because the participants need to go back even to their teenage years to provide the necessary information. Furthermore, since this is a cross-sectional study, we evaluated the association between factors and quality of life. Still, we could not evaluate these factors’ impact on change in the quality of life over time.
